# Observing the oxidation of platinum

**DOI:** 10.1038/s41467-017-00643-z

**Published:** 2017-09-05

**Authors:** Matthijs A. van Spronsen, Joost W. M. Frenken, Irene M. N. Groot

**Affiliations:** 1 Huygens-Kamerlingh Onnes Laboratory, Leiden University, PO Box 9504, 2300 RA Leiden, The Netherlands; 2 Leiden Institute of Chemistry, Leiden University, PO Box 9502, 2300 RA Leiden, The Netherlands; 3 Present Address: Harvard University, 12 Oxford street, Cambridge, MA 02138 USA; 4 Advanced Research Center for Nanolithography (ARCNL), Science Park 110, 1098 XG Amsterdam, The Netherlands

## Abstract

Despite its importance in oxidation catalysis, the active phase of Pt remains uncertain, even for the Pt(111) single-crystal surface. Here, using a ReactorSTM, the catalytically relevant structures are identified as two surface oxides, different from bulk α-PtO_2_, previously observed. They are constructed from expanded oxide rows with a lattice constant close to that of α-PtO_2_, either assembling into spoked wheels, 1–5 bar O_2_, or closely packed in parallel lines, above 2.2 bar. Both are only ordered at elevated temperatures (400–500 K). The triangular oxide can also form on the square lattice of Pt(100). Under NO and CO oxidation conditions, similar features are observed. Furthermore, both oxides are unstable outside the O_2_ atmosphere, indicating the presence of active O atoms, crucial for oxidation catalysts.

## Abstract (web summary)

Improving platinum as an oxidation catalyst requires understanding its structure under catalytic conditions. Here, the authors discover that catalytically important surface oxides form only when Pt is exposed to high pressure and temperature, highlighting the need to study catalysts in realistic environments.

## Introduction

Platinum serves as a major component in the automotive catalyst, which oxidizes CO and residual hydrocarbons to CO_2_. In addition, NO is oxidized when the catalyst is operating in lean burn, i.e., in excess oxygen. Research into the automotive catalyst remains highly relevant due to stricter emission regulations.

To improve catalysts by rational design, it is crucial to understand the structure and chemistry of a realistic catalyst under reaction conditions. However, this is often too difficult for a technical catalyst under chemical conditions and studies of model catalysts are vital. For the Pt-based automotive catalyst, the most essential model is the Pt(111) single-crystal surface. This surface has the lowest surface energy and is expected to form the largest facets in a real catalyst^1^.

The interaction of O_2_ with Pt(111) has been extensively studied under traditional surface science conditions, i.e., ultra-high vacuum (UHV). It was found that O_2_ binds molecularly below 160 K^2, 3^, above which it dissociates readily and forms a p(2 × 2)-O chemisorption overlayer with a saturation coverage of 0.25 ML. High-temperature exposure^4^ or exposure to stronger oxidants, such as NO_2_^5^, O_3_^6^, and atomic oxygen^7, 8^, was needed to create higher O coverages. This included a surface oxide consisting of one-dimensional (1D) oxidic rows, which were forming honeycomb-like superstructures^5^. Using these harsh conditions, even PtO_2_ could be created^9, 10^.

There is no guarantee that the structure of a catalyst observed in UHV is the same as the structure present under reaction conditions. This structure can only be elucidated when it is probed in situ, i.e., under high-pressure and elevated-temperature conditions. Two independent in situ surface X-ray diffraction (SXRD) studies showed the formation of bulk-like α-PtO_2_^11, 12^. These observations were contradicted by a near-ambient-pressure (NAP) X-ray photoelectron spectroscopy (XPS) study^13^, which showed the formation of a surface oxide at similar temperatures as in the SXRD experiments, but at lower pressures. This surface oxide was found to be an intermediate in the bulk oxidation of Pt, which only started at much higher temperatures. In a recent NAP XPS study, it was found that prolonged exposure to oxidizing conditions was needed to form Pt oxide^14^.

The most important questions remain unanswered. What is the structure formed under catalytically relevant conditions? If it is an oxide, is this a surface or bulk oxide?

In this work, the oxidation of Pt(111) is probed with O_2_ pressures of 1–5 bar and at 300–538 K using in situ scanning tunneling microscopy (STM). Interestingly, the formation of α-PtO_2_ is not observed, instead two stable surface oxides form. The first has a structure in which equilateral triangles are arranged into spoked wheels. The lattice constant within the spokes is close to that of α-PtO_2_. The second structure consist of a pattern of rows which are lifted from the surface and consisted of nearly half the amount of Pt atoms in the top layer. These surface oxide are not stable without the high O_2_ pressure indicating that the O atoms in these structures are very reactive, making them relevant for catalysis.

## Results

### Spoked-wheel superstructure

**Fig. 1:**
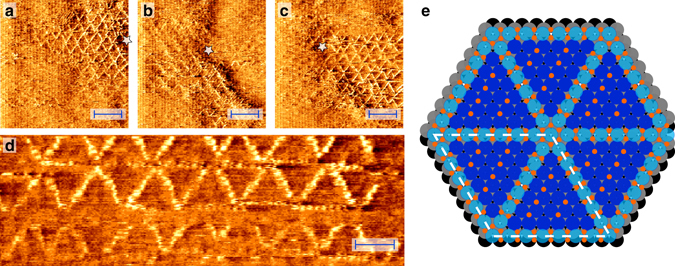
Surface oxide on Pt(111). Surface oxide with spoked-wheel structure formed on Pt(111) while exposing it to 1 bar O_2_ at 529 K. Three consecutive (**a**–**c**), large-scale STM images displaying extended domains. Field of view was slowly changing due to thermal drift (see *white star* as reference point); Enlarged detail (**d**), illustrating the atomic resolution within the spokes. *Scale bars* represent 4 nm (**a**–**c**) and 2 nm (**d**). Proposed ball model (**e**) showing the spoked wheels within, but lifted from, the surface layer (oxidized Pt, *light blue*; O, *orange*; Pt surface layers, *blue*, *gray*, and *black*). Positions of O atom were based on previous experiments and theory^5, 26^

Upon exposing a Pt(111) surface to 1.0 bar O_2_ at ~530 K, triangular features appeared on the surface, assembling into spoked-wheel superstructures, see high-pressure STM images in Fig. 1. The average length of the edges was 2.2 ± 0.1 nm, corresponding to 7.9 ± 0.4 Pt(111)-lattice constants. Domains of this superstructure extended tens of nanometers, as can be seen in Fig. 1a–c. These stable structures were still containing defects and disorder. This included translational defects, i.e., the shift of a row of spoked wheels, the incorporation of smaller triangles, and, incidentally, doubling/tripling or missing of spokes. These defects are depicted in Supplementary Figs. 1 and 2 of Supplementary Note 1. During the growth phase, lasting 17–50 min, these defects were more abundant and also a transient, distorted-hexagonal structure was observed (Supplementary Fig. 3 in Supplementary Note 2).

Several images, such as Fig. 1c, d, show clear atomic resolution within the rows. The measured atomic periodicity in the rows was 0.30 ± 0.01 nm, which was significantly larger than the Pt(111)-lattice constant of 0.278 nm and close to that of the UHV-stable surface oxide^5^, α-PtO_2_^15–19^, and PtO^15, 19, 20^. Based on this agreement, we consider this structure as a surface oxide comprised of 1D oxide rows. Most spokes consisted of eight atoms, including both endpoints, with very few being extended to nine or more. Given the spoke length of 2.2 ± 0.1 nm, this would lead to a lattice constant within the row of 0.31 ± 0.01 nm, close to, but slightly larger than the directly measured interatomic distance. As a consequence, the spoked-wheel structure had a unit cell close to (8 × 8) with the Pt atoms in the oxide rows showing an expansion of ~8/7.

This spoked-wheel superstructure was never identified before at the low pressures of most other Pt(111)-oxidation studies, nor predicted by theory. Nevertheless, related surface oxides were found earlier, obtained in an artificial way, by oxidation with stronger oxidants. On Pt(111), one of these structures resembled the spoked-wheel structure and was formed by exposing the surface to an O beam^21^. Another structure, with expanded chains assembling into honeycombs, was observed after exposure to NO_2_ at 450 K in vacuum^5^. In addition, related structures were proposed for Pt^22, 23^ and (110)-type steps^24, 25^. Although all these structures resemble some aspects of the spoked-wheel oxide, our experiments show that UHV oxidation with stronger oxidants does not guarantee to yield catalytically relevant structures.

Based on the STM measurements, the model in Fig. 1e is proposed. In this model, the O atoms in the chain are forming square planar units, surrounding a Pt atom. This geometry is based on the DFT calculations by Hawkins et al.^26^ and are structurally related to PtO and Pt_3_O_4_ with which they share the square planar PtO_4_ motifs. The spoked wheels are not on top of the surface but within the top layer, from where they are slightly lifted. This conjecture is based on the apparent height of the spokes that is much lower than a mono-atomic Pt step (Supplementary Table 1 in Supplementary Note 3) and is consistent with previous experiments^5^ and DFT calculations^26–28^. The O coverage of the proposed model is 0.75 ML (Supplementary Note 4), three times more than generally obtained by O_2_ exposure in UHV^2, 3, 29–31^.

The oxidation experiments were also performed using the square Pt(100) surface, which has a quasi-hexagonal reconstruction under UHV. Under oxidizing (1 bar O_2_ and ~530 K) conditions, the surface is predominantly covered with a network of rectangular islands, which consisted of rows with a double periodicity (Supplementary Fig. 4a). However, triangles were observed on several occasions (Supplementary Fig. 4b, c). This could indicate that the surface locally reconstructed back into a (quasi)hexagonal structure.

### Large-scale formation of islands

**Fig. 2:**
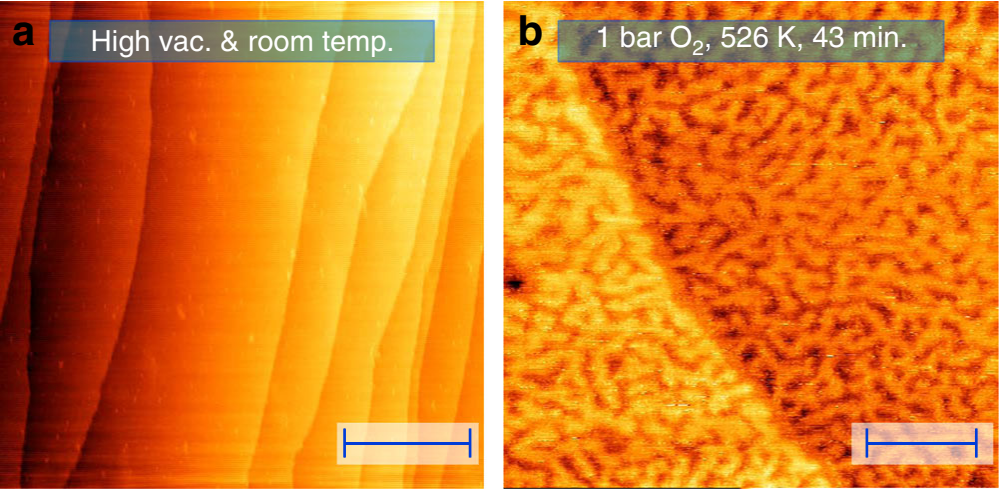
Larger-scale structure of the surface oxide. STM images showing the change in large-scale morphology upon high-pressure O_2_ exposure. Freshly prepared Pt(111) surface (**a**) in the reactor (high vacuum at room temperature); stable, oxidized surface (**b**) at 1.0 bar O_2_ and 531 K after 43 min exposure to these conditions. Nonlinear filtering was applied to remove artifacts due to vibrational interference of the microscope. *Scale bars* represent 100 nm (**a**) and 20 nm (**b**)

Parallel to the formation of the spoked-wheel oxide, the surface roughened in a well-defined manner (compare Fig. 2a, b). The first shows the surface in the vacuum of the reactor prior to high-pressure exposure. Figure 2b shows an in situ STM image of a surface region, exhibiting a mono-atomic step from the top left to the middle of the lower edge. Hence, the surface consisted of two terrace levels. Within each terrace, the surface showed a complicated network of worm-shaped islands.

The islands divided each terrace in two levels. Figure 2b suggests that the top level of the lower terrace was at the same height as the lower level of the upper terrace. In other words, the image indicates that the island structure divided each terrace over two height levels of the Pt(111) surface. This was supported by the depth of the voids in between the islands, which was 0.21 ± 0.02 nm, agreeing well with a mono-atomic Pt step. Therefore, we conclude that solid, flat terraces transformed into networks of mono-atomic-high, worm-shaped islands upon O_2_ exposure. Through the voids, the lower lying terrace became visible. This resulted in a two-level-terrace structure. We interpret this large-scale roughening as the consequence of the stress exerted by the oxide on the surface. This stress was relieved by forming the two-layer network. Interestingly, domains of the spoked wheels extended over several islands, including the voids in between.

### Increasing O_2_ pressure

**Fig. 3:**
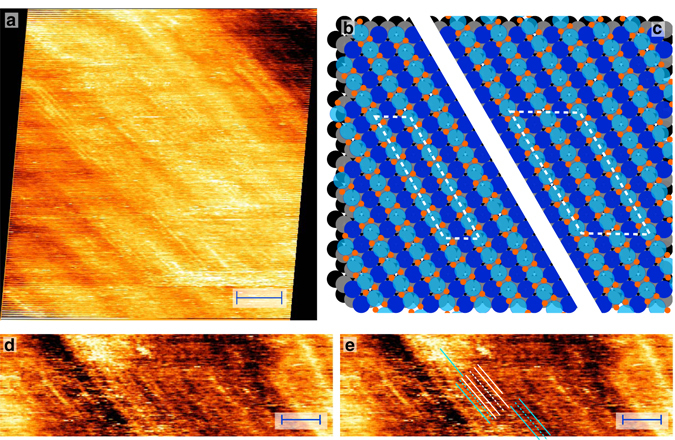
Structures at higher O_2_ pressure. Lifted-row structure formed at higher O_2_ pressure (observed above 2.2 bar). STM image (**a**) recorded at 535 K in 3.8 bar O_2_, and 98 min in the experiment, while increasing the total pressure from 2.7 to 6.0 bar. The image is shown after drift correction and some nonlinear filtering to remove a few horizontal scan vibrations; Ball model of two examples of the lifted-row oxide in which the O atoms are ordered differently: lifted rows are in phase (**b**) resulting in straight lines of O atoms and the smallest O–O separation; largest O–O separation (**c**), due to shifting of adjacent rows by half a period. These structures have a (2 × 8) and c (4 × 8) unit cell, **b** and **c** respectively; Two replicas of an enlarged detail (**d**, **e**) of the lifted-row structure. Indicated lines (**e**) show several interesting features: missing rows, a light domain wall, and point defects. (Shown without drift correction but with a modest amount of nonlinear filtering to remove a few horizontal scan vibrations.) *Scale bars* represent 4 nm (**a**) and 3 nm (**d**, **e**)

After a stable spoked-wheel structure was formed (1.0 bar, 530–538 K), the O_2_ chemical potential was increased by gradually raising the O_2_ partial pressure to 5.0 bar. Starting at 2.2 bar O_2_, a new structure was observed (Fig. 3a). The image shows a pattern of parallel rows, which we will refer to as a lifted-row structure. Importantly, the spoked-wheel structure was still observed at all the experimentally probed O_2_ pressures. The domains of both structures were rather large and estimated to be above 100 nm. Different rotational domains of the lifted-row structure were observed with an approximated size of, again, above 100 nm. This was concluded from the absence of boundaries separating rotational domains.

Because the pressure was increasing, thermal drift was quite strong and highly nonlinear. The drift correction, based on the overlap between consecutive images, was difficult to perform accurately due to the, mainly, 1D character of the morphology and the presence of considerable dynamics. After correction, the row-to-row distance was 0.46 ± 0.01 nm (0.44 ± 0.01 nm before correction). This is close to $\sqrt 3$ × *a* (*a* = nearest Pt–Pt distance), which equals 0.48 nm, making this surface oxide commensurate with the Pt(111) substrate. Within the rows, the STM images revealed weak atomic features. However, these were not ordered enough to reliably determine a lattice constant. The measured height corrugation across the rows was very modest with an average value of 0.03 ± 0.01 nm, roughly a tenth of a mono-atomic Pt step.

A model for the lifted-row oxide with similarly expanded oxide rows as the spoked-wheel oxide is depicted in Fig. 3b, c. The O coverage in this model is 0.88 ML (Supplementary Note 4). The O atoms of neighboring rows can be disordered or ordered. Two ordered examples are shown in Fig. 3b, c. In the first (Fig. 3b), the lifted rows are ‘in phase’, minimizing the O–O separation between adjacent stripes and is expected for attractive O–O interaction. In the second example (Fig. 3c), the O–O separation is maximized by shifting the phases between adjoining rows, expected for repulsive O–O interactions. The unit cells for these two configurations are (2 × 8) Fig. 3b (4 × 8), Fig. 3c, respectively.

Two translational domains were observed, since either the odd or the even row could be lifted. They are indicated by the *white* and *light blue lines* in Fig. 3e (annotated replica of Fig. 3d). The *dashed lines* correspond to missing rows. In some cases, those missing lines were defects within a translational domain, while sometimes they were boundaries (so-called light domain walls) between two translational domains. In addition, point defects were observed, in which a single row switched domain.

### Effect of temperature

Exposing the Pt(111) surface to 1.0 bar O_2_ at lower temperature (291–338 K), did not lead to large-scale ordered structures, but did lead to disordered structures. At these lower temperature conditions, thermodynamics predicts an even higher driving force to incorporate O. This seems to contradict the observations and, hence, two possibilities remain. First, the disordered structures did contain more O than the ordered surface oxides. In this case, the structures would have to be disordered multilayers or contain subsurface O. Second, the formation of the ordered surface oxides was kinetically hindered. The latter notion is supported by the low-temperature observation of the basic structural elements, lifted rows and triangles, that are the building block for the ordered, higher temperature structures (Supplementary Fig. 5 in Supplementary Note 5). The limiting step can either be the rearrangement of the Pt atoms on a larger scale or the dissociation of O_2_. Similar observations were made after oxidation of Pt(110) at lower temperature^22^.

The main features observed at lower temperature were clusters with a diameter of 0.32 ± 0.04 nm. Their apparent height was much smaller than a mono-atomic step and was unaffected by changes in the sample bias. Therefore, we propose that these clusters were single PtO_*x*_ units, slightly lifted from the surface (Supplementary Table 2). It could be the building block of both the spoked-wheel and the lifted-row surface oxides. Similar clusters were previously observed^5^ and are possibly PtO_3_ units, as proposed by DFT^26^.

### Vacuum stability of the surface oxides

The adsorption strength of the O atoms in the surface oxides was evaluated by evacuating the reactor after in situ growth at high-pressure, elevated-temperature conditions. The decrease in pressure was rapid, reaching the (U)HV-regime within minutes, at which point the STM imaging was resumed (Supplementary Fig. 6 in Supplementary Note 6). At the same time, heating of the surface was stopped, allowing the sample to cool down. After evacuation, no large-scale ordered structures were observed. In addition, the surface was very dynamical, leading to tip-induced restructuring and noisier STM images. In these experiments, no distinct structures, surface oxides nor chemisorption structures, were observed. This strongly suggested that the surface oxides were not fully stable upon evacuation, with oxygen (slowly) desorbing or reacting with residual gas.

### Coverage estimate

**Fig. 4:**
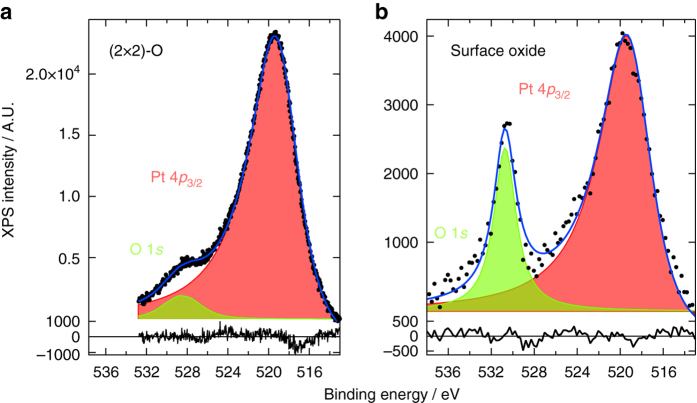
XPS after oxidation. XP spectra collected after exposure of a clean Pt(111) sample to 400 L O_2_ at room temperature (**a**) and to 1.0 bar O_2_ at 441–444 K for 60 min (**b**). Spectra were fitted with a Doniach–Šunjić function convoluted with a Gaussian: O 1 s (*green*) and Pt 4*p*_3/2_ (*pink*). Lower panels show the fitting residual. To obtain a good fit, the Pt 4*p*_3/2_ peak required considerable asymmetry in both spectra

XPS measurements in vacuum after high-pressure, elevated-temperature exposure yielded a coverage estimate of 0.9 ± 0.1 ML, after comparison to the saturated *p*(2 × 2)-O chemisorption structure of 0.25 ML^2, 3, 29–31^ (Fig. 4). In addition, the binding energy was shifted from 528.5 eV, lower than typically observed for O(ads)/Pt (Supplementary Table 3), to 530.7 eV. The higher binding energy agreed well with the values found for oxidized Pt (Supplementary Table 4), although a clear trend in the literature is missing. A second XPS study (Supplementary Fig. 7a in Supplementary Note 7) focused on the decomposition of the surface oxide. The O coverage showed a constant, almost linear decrease with increasing temperature (Supplementary Fig. 7b). Although desorption started above 432 K, some O was still present at 963 K. This is either above or on the higher edge of O_2_ desorption from the Pt(111), Pt(100), and Pt(110) surfaces^2–6, 8, 22, 32–36^. The increased stability could have resulted from the surface roughness^37^ or from subsurface O^37, 38^.

The binding energy was remarkably constant during decomposition of the oxide (Supplementary Fig. 7b). The absence of a binding energy shift can be explained by an area reduction of the surface oxide coexisting with bare, metallic Pt. Alternatively, it could have decomposed into a chemisorption structure, but only if that was rather different from the *p*(2 × 2)-O structure obtained in UHV, as the O 1*s* binding energy did not shift from that of the surface oxide. As with the temperature stability, this can be related to oxygen-induced roughening and is supported by the work of Parkinson et al.^7^, in which a (2 × 2) diffraction pattern was observed on Pt(111) during oxide decomposition^7^. The (2 × 2) state had an O 1*s* binding energy of 530.8 eV, very close to our reported values.

### Stability with respect to α-PtO_2_

In this study, α-PtO_2_ was not observed, contradicting two SXRD studies on the same surface under similar conditions^11, 12^. The conclusions in these studies were strongly based on the in-plane diffraction spots, which indicated a hexagonal overlayer with a lattice constant close to that of α-PtO_2_. However, the spoked-wheel oxide would give similar in-plane hexagonal diffraction spots. Furthermore, both studies found a hexagonal unit cell parallel to the Pt(111) unit cell. However, α-PtO_2_ would be expected to have a 30° rotation with respect to the substrate^1, 9, 10, 39^. On the other hand, X-ray-induced O_3_ formation could also explain the discrepancy^12, 40^. Ozone was observed to strongly oxidize Pt even under UHV conditions^6^.

Calculated phase diagrams show that α-PtO_2_ should be the stable phase under the experimental conditions^1, 39, 41^, suggesting that the surface oxides were only metastable with respect to α-PtO_2_. In this case, there should be a strong kinetic limitation to form α-PtO_2_, because the surface oxides are fully stable in our experiments. Since significant mobility was observed, any limitation should be related to O_2_ dissociation. In a NAP XPS study^13^, α-PtO_2_ was observed only at 720 K. More important than thermodynamic stability is the question of stability under catalytic conditions.

### NO and CO oxidation conditions

The surface oxides observed in this study were unstable in vacuum. From the instability of the surface oxides in the evacuation experiments, it can be concluded that these oxides contain O atoms that are loosely bound and therefore highly reactive. For the spoked-wheel oxide, an interesting question to answer is which O atoms are more reactive. Are those the O atoms forming the Pt oxide stripes or are those the chemisorbed O atoms, which in turn can feel a strong repulsion from the Pt oxide spokes?

The spoked-wheel oxide developed similar roughness as that observed under CO oxidation conditions in an O_2_-rich flow^42^. Under these conditions, a higher reactivity was found for CO oxidation. Both the roughness and the higher reactivity phase can very well be explained by the formation of a single layer of the spoked-wheel oxide. In this case, CO can react with the O atoms of the surface oxide via a Mars-van Krevelen reaction mechanism. Similar large-scale structures were observed under NO oxidation conditions (Supplementary Fig. 8). Similar CO oxidation studies under reaction conditions for Pt and Pd model systems have recently been reviewed^43, 44^ and the explanation of surface oxides as an active phase in oxidation reaction is in line with results for, e.g., the Pt(110)^45–48^ and Pd(100)^49–51^ surfaces.

## Discussion

The STM images with the highest resolution were obtained with low bias voltages (10–20 mV), implying that the surface oxides had no or a very small band gap. Although conflicting with observed band gaps of the surface oxide and oxide clusters on Pt(110), a small or lacking band gap is not fully unpredictable. Pt oxides have relatively small band gaps. For example, experimental values for crystalline PtO_2_ were 1.8^52^ and 2.5 eV^53^ and for PtO it is not fully clear whether it even has a band gap^19, 20, 54–56^. Furthermore, structural disorder lowers the band gap^57, 58^.

The observed 1D oxide chains are structurally more related to the lower band-gap oxide, PtO. In addition, the Pt atoms in the 1D oxide chains are closely spaced with those not directly involved in the surface oxide and, possibly, these two kinds of Pt atoms can sufficiently bind and eliminate the band gap. Alternatively, the absence of a band gap can be rationalized by a fractional oxidation state. Oxidation states of Pt oxides deviate strongly from the formal oxidation states of Pt^2+^ and Pt^4+^, having significant covalent character^24, 26, 55, 56, 59, 60^, which can also be expected for the surface oxides. This fractional oxidation state results in partial occupation of the highest *d*-orbital. Finally, the abundantly present defects in these surface oxides can also explain the lack of band gap.

Exposing the Pt(111) model catalyst to oxidizing atmospheres under realistic catalytic conditions (300–538 K, 1–5 bar O_2_) leads to the formation of two different single-layer surface oxides, the spoked-wheel and the lifted-row oxide. We propose that both consist of expanded, Pt oxide rows. Ordered surface oxides were solely observed under both high pressure and elevated temperature, proving the absolute necessity of in situ measurements. Moreover, these surface oxides were not stable without the presence of the high O_2_ pressure. Additional experiments indicated that these surface oxides are important for CO and NO oxidation. Furthermore, similar structures are formed on Pt(100).

To translate the results obtained on a Pt(111) model catalyst to real, complex catalysts, one important point is the effect of nano-confinement. This confinement is a direct consequence of the use of nanoparticles as catalysts. For the smallest particles, their (111) facets will be on the same order of size as the spoked wheels. This could make the spoked-wheel oxide less stable. On the other hand, nanoparticles may have less difficulty to relieve the strain induced by the surface oxide than extended single crystals. To clarify this point, in situ experiments yielding atomic resolution on the facets of particles, possibly aided by DFT calculations, will be needed.

## Methods

### ReactorSTM

All experiments were performed in a ReactorSTM^61, 62^, which is a UHV system housing a small-volume (0.5 ml) flow-cell reactor integrated with an STM. The design is such that all delicate STM parts are outside the reactor and not exposed to gases or to high temperatures. Only the polished side of the sample and the tip with its sliding tip holder are inside the reactor. To seal the high-pressure reactor from the UHV chamber, a fluoroelastomer seal^63^ is used. In this way, UHV conditions are maintained in the surrounding chamber, even when the reactor pressure reaches 6 bar. The fluoroelastomer limits the temperature of the high-pressure experiment to 600 K. The temperature is measured with a K-type thermocouple. The sample is heated with a filament placed on the vacuum side of the sample. In addition, the system houses equipment for low-energy electron diffraction, Auger electron spectroscopy, and XPS, which can be employed before and after high-pressure exposure of the sample.

### Single-crystal sample preparation

The platinum single crystal (Czochralski-grown, purity of 5N^64^) was polished to the (111) plane within 0.1°. After polishing, it was cleaned by Ar^+^ sputtering (~20 min, beam energy of 1.0 keV, and sample current of 3–4 μA) and annealing both in O_2_ (~2 min at 948 K in 1 × 10^−7^ mbar O_2_) and in UHV (at 1130 K for ~10 min). At least 60–70 of these cycles were performed before starting the first experiment and around 5 cycles were completed between consecutive experiments. The preparation method was routinely checked with both LEED (Supplementary Fig. 9) (Omicron SPECTALEED, 4 grid) and AES (Omicron SPECTALEED, retarding field analyzer).

### Gases

The gases (O_2_: Praxair 5.0N and CK 4.5N and Ar: Linde 6.0N, Westfalen 5.0N, Praxair 5.0N) were used without further purification. Unless specified otherwise, all experiments were performed at a total normal flow, i.e., the flow at a pressure of 1.013 bar at 273 K, of 10 ml/min with 83.3% O_2_ and 16.7% Ar.

### STM measurements

STM measurements were performed with cut PtIr tips and in constant-current mode using a video-rate STM controller^65, 66^. Both planar and linear line-by-line background subtractions were employed. In order to emphasize local height variations, several images are presented in differentiated form with the color indicating the local slope measured from left to right. To calibrate the piezoelectric scan element, atomically resolved Au(111) STM images were used. These images were recorded with a closed reactor, with a seal in place at room temperature, in vacuum. This resulted in a calibration with a standard deviation of 1.5%.

### XPS measurements

All XP spectra were collected in UHV, but without exposing the sample to air, using Al K*α* radiation (VG XR3E2, non-monochromatic). Emitted photoelectons were detected with a hemispherical analyzer (VG Clam2/1VU, 100 mm radius) equipped with a collimator aperture to bring the spot size down to ~8 mm. The detector was operated with a slit size of 4.0 mm and the electrostatic lenses at 3:1 mode, reducing the spot size further to ~1.3 mm. The sample was facing the detector, with the X-ray beam impinging under an angle of 55° with respect to the surface normal. To ensure that the inspected area coincided with that exposed in the reactor, the ring-shaped imprint of the fluoroelastomer was used, on which a strong F peak could be discerned. Importantly, the center of the sample did not show any F contamination before or after the high-pressure experiments. The energy range of interest, around the O 1*s* and Pt 4*p*_3/2_ peaks, was probed with a constant analyzer energy (25 V). The binding energies were calibrated against the known energy of the Pt 4*p*_3/2_ peak of 519.5(3) eV^67^. After calibration, the spectra were corrected by subtracting a linear background. The XP peaks were fitted with a Doniach-Šunjić function^68^ convoluted with a Gaussian, requiring the following fitting parameters: binding energy, intensity, Lorentzian line width, Gaussian line width, and asymmetry factor. For the temperature-dependent experiments, a simpler procedure was followed, consisting of subtraction of a constant background and normalization to the maximum Pt 4p_3/2_ intensity. This was followed by fitting the O 1*s* peaks with a Gaussian function.

### Data availability

The data that support the findings of this study are available from the corresponding author upon reasonable request.

## Electronic supplementary material

Supplementary Information

Peer Review File
