# Supplementary material for: Observing the oxidation of platinum
Source: Nat Commun. 2017 Sep 5;8:429. doi: 10.1038/s41467-017-00643-z (PMC5585323; doi:10.1038/s41467-017-00643-z)
Supplement: Supplementary file 1 — Supplementary Information [file 41467_2017_643_MOESM1_ESM.pdf]

Description of Supplementary Files

File Name: Supplementary Information
Description: Supplementary Notes, Supplementary Figures, Supplementary Tables, and Supplementary References.

File Name: Peer Review File

Supplementary Note 1: Defects

Under the high-pressure O_2 conditions, the spoked-wheel oxide formed large domains all across the surface. However, the formed structures were not perfect and contained many defects. Some of these defects occurred only during growth, while others persisted in the stable structures. These defects can be seen in Fig. 1 of the main text.

The presence of these defects became clear when the fast Fourier transform (FFT) of Fig. 1d was compared to the calculated FFT for a perfect spoked-wheel pattern (inset of Supplementary Fig. 1b). These FFTs are depicted in Supplementary Figs. 1a and b, respectively. The FFT of the perfect spoked-wheel structure (Supplementary Fig. 1b) is a 30° rotated spoked-wheel pattern. The spokes are comprised of individual higher order spots reflecting the distance between parallel spokes. These spots were also clearly visible in the experimentally obtained pattern (Supplementary Fig. 1a). However, the experimental spots were elongated in the vertical direction.

This vertical streakiness indicated that the structure of Fig. 1d exhibited a translational defect that divided the pattern in two translational domains. This is visualized in Supplementary Fig. 1c, which depicts an FFT of a spoked-wheel pattern containing a horizontal translational defect. More specifically, one diagonal (top left to bottom right) was streaked symmetrically while the other (top right to bottom left) was asymmetrically elongated, alternating in top and down direction. This asymmetric behavior was also visible in the experimental FFT. Evidence of these translational defects was frequently observed in the scanning tunneling microscopy (STM) images, for example in Supplementary Fig. 2a and the corresponding model (Supplementary Fig. 2d).

In addition to translational defects, also other structural defects were observed. Most prominently, the incorporation of several smaller triangles in the structure. This is illustrated by Supplementary Fig. 2b and e. This figure shows that smaller triangles clustered. These clusters need to occupy the same space as an integer number of regular triangle(s) in order for the structure to remain close packed without voids. The smaller triangles had a minimal edge length of 1.3 nm or 4–5 Pt(111)-lattice constants.

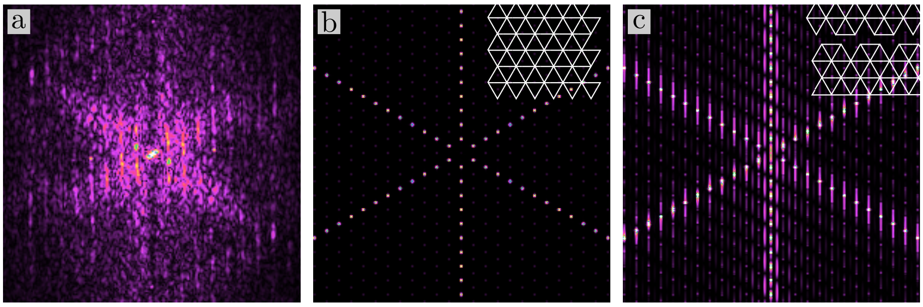

Supplementary Fig. 1: FFT analysis of structural defects. FFT (a) of STM image showing the spoked-wheel structure (Fig. 1d), FFT (b) of perfect, extended spoked-wheel structure, and FFT (c) of spoked-wheel structure with a translational defect creating a horizontal defect line. Insets show the respective structures from which the FFTs were calculated.

A third class of defects was the doubling or tripling of spokes. Similar defects were reported by Devarajan et al.[1] and Weaver[2] for the previously reported surface oxides. However, confirming the existence of multiple spokes can be difficult by STM. The images can appear heavily distorted when the tip does not end in a single apex. In this case, the STM images are comprised of several overlapping replicas, translated by the distance between the tip apexes, leading to double or triple spokes. Generally, double spokes were not frequently occurring in stable structures. In the early stages of growth, this defect was commonly observed. One example is presented in Supplementary

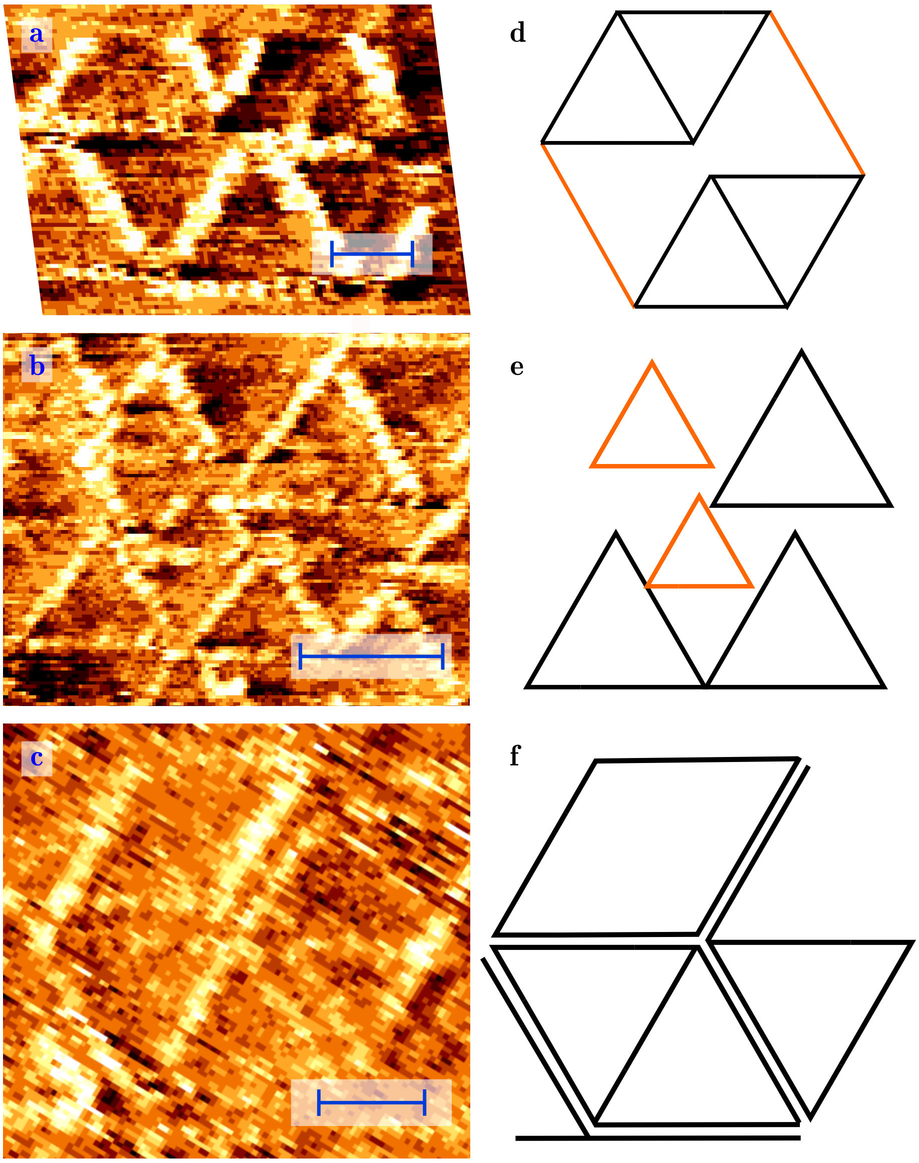

Supplementary Fig. 2: Structural defects. STM images with structural models showing various structural defects (depicted by orange lines), such as translational defects (a and d), incorporation of smaller triangles (b and e), and doubling and missing of spokes (c and f). All STM images were obtained at $p_{O_2} = 1$ bar and $T = 529$ K, $U_{bias} = 0.05$ V, $I_{tunnel} = -(0.89–0.98)$ nA, and the exposure times were 31, 33, and 18 minutes, respectively. The STM images are shown after line-by-line differentiation. Image c was recorded with a different scan orientation and rotated 31° to match other STM images. Scale bars represent 2 nm (a and b) and 1 nm (c).

Fig. 2c and f. In this case, an STM-tip artefact could be excluded. This was because doubling of spokes was observed in all three directions, but not all spokes were doubled, which would be expected if imaged with a multiple tip. This image also illustrates that doubling of spokes usually concurred with missing spokes.

The separation between the spokes in Supplementary Fig. 2c was equal to 0.38±0.03 nm. The error bar was rather high but not related to the thermal drift, which was estimated by following large-scale morphology movement and was at its maximum 1% of the image dimensions. The calculated separation was almost equal to the row-to-row distance of the transient, distorted-hexagonal structure that will be discussed in the next section.

Supplementary Note 2: Transient, distorted-hexagonal structure

The initial, dynamic stage is summarized in Supplementary Fig. 3a, which shows a high-resolution STM image of typical transient structures. In this STM image, several triangles could be noticed in a few locations and they were starting to form spoked wheels. In addition to these emerging spoked wheels, a row-like structure could be distinguished. This structure, in fact, showed a hexagonal-like symmetry in the highest resolution regions of the image (see regions close to the arrows, Supplementary Fig. 3a).

A perfect hexagonal lattice can appear as rows in one of the directions of the lattice due to a minor asymmetry in the tip apex, i.e., the STM tip is not as sharp in one of the spoke directions as in the other two. This asymmetry results in a somewhat poorer resolution and the image appears to have rows in that direction. However, in Supplementary Fig. 3a this was definitely not the case, since the upper region of the STM image showed three rotational domains (blue, black, and green arrows) within single scan lines. For this to be an STM-tip artefact, the STM tip should have changed its apex several times per scan line at more or less the same positions. This is highly unlikely, even more since the STM conditions were such that very stable STM feedback was obtained in this part of the experiment.

The second explanation for the observation of these rows requires anisotropy in the observed lattice, i.e., different lattice constants in different directions. If these lattice constants are in the order of the best attainable resolution of a particular STM tip, then the direction of the smallest lattice constant is not resolved completely and the structure appears as rows. This occurs even with a perfectly symmetric STM tip. This means that the observed lattice had, in fact, a distorted-hexagonal structure, in which the lattice was expanded perpendicular to the rows. The three rotational domains were all distinguished in Supplementary Fig. 3a (blue, black, and green arrows).

The distorted-hexagonal structure was characterized by studying the FFT of the region indicated by the white square in Supplementary Fig. 3a. The FFT image is represented in Supplementary Fig. 3b. It clearly shows the distorted-hexagonal pattern. The pattern was compressed along the white line indicated in Supplementary Fig. 3b. This compression in reciprocal space is equivalent with an extension in real space. The other rotational domains showed similar but rotated patterns. Therefore, the distortion could not be explained by thermal drift, because that would have affected all regions equally as they were imaged in the same time interval.

Supplementary Fig. 3a shows atomic resolution both perpendicular and parallel to the rows. The lattice constant within the rows was determined by measuring the corresponding spots in the FFT image. A lattice constant of 0.30±0.01 nm was found, which corresponded well to the in-spoke lattice parameter of the spoked-wheel structure, the previously observed surface oxide[1], and the lattice constant of α-PtO_2[3–7] and PtO[3,7,8]. The average distance between the rows was determined directly from height profiles

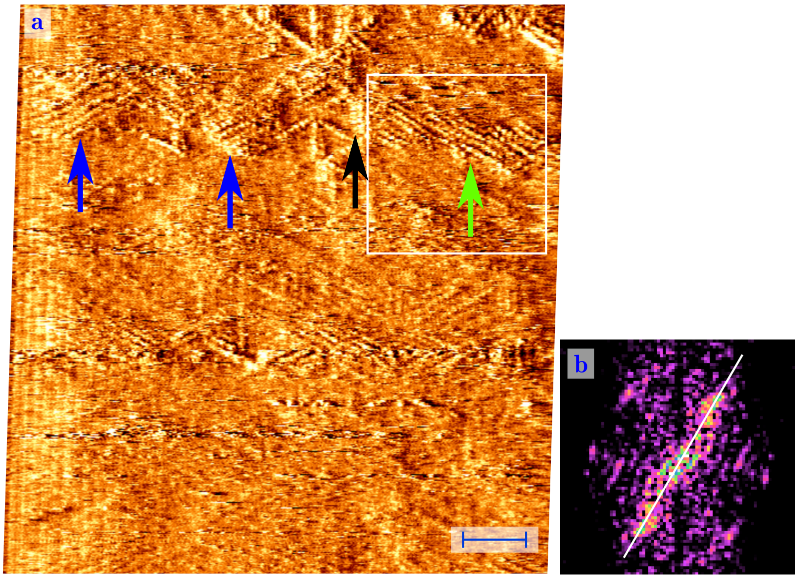

Supplementary Fig. 3: Transient oxides. STM image (a) showing the transient structures observed before a stable spoked-wheel structure had developed on a large scale (25×26 nm^2, $U_{\mathrm{bias}}=0.05$ V, $\overline{I_{\mathrm{tunnel}}}=-1.01$ nA, $t_{\mathrm{image}}=8.4$ s, line-by-line differentiated). Surface was exposed to 1.0 bar O_2 at a temperature of 527 K for 18 minutes. Colored arrows indicate different rotational domains of the distorted-hexagonal structure. For the region in the white square, the FFT (b) was calculated. The white line indicates the axis of reciprocal-space compression of the hexagonal structure. Scale bars represent 3 nm.

across several rotational domains. A value of 0.37 ± 0.04 nm was found, which was midway between 0.24 nm and 0.48 nm ($\frac{1}{2}\sqrt{3}$ and $\sqrt{3}$ times the Pt(111) lattice constant). So, the distorted-hexagonal structure was nonepitaxial with respect to the Pt(111) lattice.

Minute patches of this structure remained present in the stable spoked-wheel structure. This can be seen in Fig. 1a (main text). The quality of that STM image was not good enough to resolve the atomic structure within the rows, but the distances between the rows matched that of the distorted-hexagonal structure.

This structure was different from the lifted-row structure, discussed in the main text. It differed in two important ways, namely, it was not a stable structure and the average distance between the rows was much smaller than that of the lifted rows of the lifted-row structure.

The Pt content of this structure was around $\frac{0.28}{0.30}\times\frac{0.24}{0.37}\approx 0.61$ compared to a Pt(111) ML. We had no information on the position and number of O atoms in this structure. If we would assume a PtO_2 composition, we would derive an O coverage of around 1.2 ML. This would be higher than the O coverage of the stable surface oxides. This higher O content of the transient structure with respect to the stable surface oxides seems counterintuitive, since it would exceed the O coverage that the surface acquired at even higher O_2 pressures. The structure could also have had a composition of one of the other Pt oxides, such as Pt_3O_4,[6,9–12], Pt_2O[13], or Pt_5O_6[10,11]. In view of these uncertainties, we will not speculate further on the O content of this structure. Furthermore, it remains unclear why this structure was anisotropically expanded, but it could be related to stress relief. After initial relaxation in one direction, the resulting stress might be too low to induce similar relaxation in the other two directions.

Supplementary Note 3: Apparent height of spokes

The apparent height of the spokes of the spoked-wheel structure was measured at three different bias voltages (10–100 mV) with the same tip in the same experiment. The bias range was small due to the fact that low positive voltages were required to obtain atomic resolution of the spoked-wheel structure. The apparent height showed a small variation with bias voltage as presented in Supplementary Table 1, but the effect is very small with respect to the error bars.

A second measurement at 0.05 V in a different experiment showed much less height corrugation, i.e., 0.03±0.01 nm. This was only about one third of the corrugation that was found in the first measurement at the same bias voltage. Both experiments were performed with the same PtIr tip, but the tip apex was altered between the experiments due to a crash in the surface. It showed that the apparent height was much more sensitive to the shape and/or state of the tip apex than to the applied tunneling voltage.

Supplementary Table 1: Apparent height of the spokes of the spoked-wheel structure observed during the exposure of the Pt(111) surface to 1.0 bar O_2 at 529–537 K at three different sample bias voltages. The second measurement at 0.05 V was performed with the same STM tip, but in a separate experiment and most likely with a different tip apex.

Bias/V	App. height/nm	Stand. dev./nm
0.10	0.12	0.06
0.05	0.11	0.04
0.01	0.10	0.03
0.05	0.03	0.01

Supplementary Note 4: Calculation of ideal O coverages

Spoked-wheel oxide The (8×8) unit cell contains 4 Pt oxide spokes on the edges of the unit cell (sharing O atoms between two adjacent unit cells) and one spoke in the inside of the unit cell. Every spoke contains 7 Pt atoms with a PtO_2 composition, thus 14 O atoms. One O atom between spokes is shared, so the six corners reduce the number of O atoms with 6. This leads to:

$$\begin{array}{rl} 4\times14\times0.5=28 & \\ 1\times14\times1=14 & \\ 6\times-1=-6 & + \\ \hline \text{36 O atoms per unit cell} & \end{array}$$

Additionally, 6 chemisorbed O atoms reside in the inner region of the triangle. So, the total number of O atoms is 48, 36+(2×6). In a (8×8) unit cell on the Pt(111) surface, 64 Pt atoms are located and this leads to $\frac{48}{64}$=0.75 ML.

Lifted-row oxide The following calculation was based on the lifted-row oxide model with the (2×8) unit cell as depicted in Fig. 1e of the main text. A calculation based on the (4×8) unit cell, would lead to the same result. The lifted-row oxide holds one row of 7 expanded Pt atoms with a composition of PtO_2. This amounts to 14 O atoms. A (2×8) unit cell on a Pt(111) surface contains 16 Pt atoms. The total O coverage equals $\frac{14}{16}$=0.88 ML.

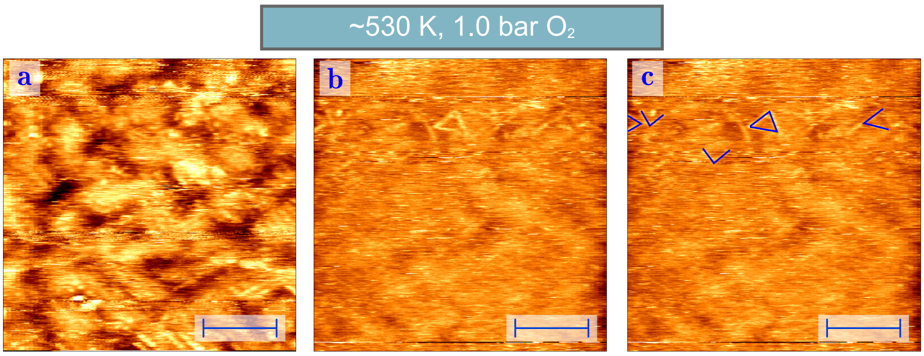

Supplementary Fig. 4: Oxidation of the Pt(100) surface. In situ STM images of the Pt(100) surface recorded at 1.0 bar O_2 and ~530 K. Predominant structure consisting of rectangular islands in which a row structure is visible (a). Some areas (b) are covered with a triangular structure (depicted in c) similar to the spoked-wheel oxide. Scale bars represent 5 nm.

Supplementary Note 5: Exposure at low temperature

To investigate the kinetics of the Pt(111) surface oxidation, experiments were repeated at lower temperatures (327–328 K and ∼291 K) at an O_2 partial pressure of 1.0 bar. These experiments did not lead to large-scale ordered structures. This absence cannot be attributed to a lower resolution, as could have been caused by a blunter tip. The acquired STM images all showed subnanometer features as illustrated in Supplementary Fig. 5.

Careful inspection revealed that the STM images obtained at lower temperatures at high O_2 pressure have recurring structural motifs. These patterns hinted at the formation of similar structures to those observed at high temperature. The predominant structure observed in the STM images (Supplementary Fig. 5a) at low-temperature exposure was a pattern of rows. The rows are indicated in the lower right panel of Supplementary Fig. 5a (annotated replica of the upper right panel). The periodicity of these rows was 0.49±0.02 nm and matched that of the lifted-row structure observed at high-temperature O_2 exposure, which was 0.46±0.01 nm. Both also corresponded well with $\sqrt{3}$ times the Pt(111) lattice constant, which is 0.48 nm.

A second observed structure was a triangular unit, the structural element of the spoked wheels, observed in several STM micrographs (Supplementary Fig. 5b) at 327–328 K. The small panels of Supplementary Fig. 5b show two duplicates of an enlarged region, in which a triangular shape was recognizable. This shape is depicted in blue in the lower right panel. Furthermore, the STM image contained many atomic features of various shapes. These features were true atomic shapes and did not originate from scan vibrations.

Finally, a third returning structure is shown in Supplementary Fig. 5c. In this STM image, several isolated clusters could be seen. Several of these clusters were analyzed as indicated in the lower right panel (annotated replica of the upper right panel). The average height between the top of the cluster and its surrounding minima was 0.06±0.02 nm (at a bias of 0.04 mV). Although this value was not fully independent of bias voltage, the variations were small and all values were well below the Pt(111)-step height of 0.23 nm (Supplementary Table 2). The average estimated full width at half maximum (FWHM) was 0.31±0.04 nm. The FWHM of these clusters agreed well with the measured lattice constant of the spoked-wheel structure.

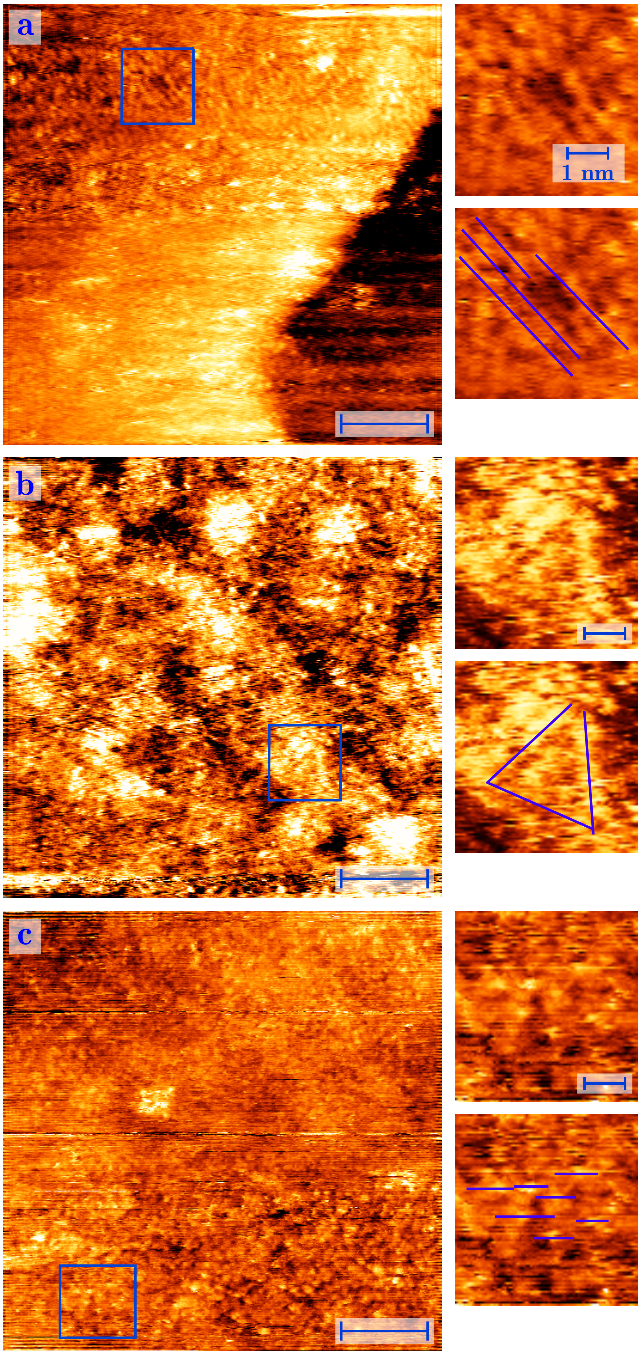

Supplementary Fig. 5: Structure observed at low temperature. STM images (a–c, 25×25 nm^2), shown together with enlarged regions from an STM dataset measured at 327–328 K at an O_2 pressure of 1 bar. Several structural features could be discerned that hinted at the formation of disordered versions of the ordered high-pressure structures found at higher temperatures: rows (a, 20 minutes exposure to O_2, $U_{bias} = -0.05$ V, $\overline{I_{tunnel}} = 0.48$ nA, $t_{image} = 9.9$ s), triangles (b, 14 minutes exposure to O_2, $U_{bias} = -0.02$ V, $\overline{I_{tunnel}} = -0.66$ nA, $t_{image} = 8.5$ s), and clusters (c, 48 minutes exposure to O_2, $U_{bias} = 0.04$ V, $\overline{I_{tunnel}} = -1.19$ nA, $t_{image} = 15.5$ s). Nonlinear filtering was applied to remove some interference. Furthermore, the right panels of image b have a nonlinear adaptive color scale to enhance contrast. Scale bar represent 5 nm (left panels) and 1 nm (enlarged regions on the right).

Apparent height of clusters

In the experiments at lower temperature (~328 K and ~291 K), clusters were observed while the surface was exposed to 1.0 bar O_2. The apparent height was measured at several different sample bias settings. The results are presented in Supplementary Table 2.

Supplementary Table 2: Apparent height at three different sample voltages of clusters observed during the exposure of the Pt(111) surface to 1.0 bar O_2 at lower temperatures (327–328 K).

Bias/V	App. height/nm	Stand. dev./nm
0.69	0.09	0.02
0.04	0.06	0.02
-0.05	0.12	0.04

Supplementary Note 6: Evacuation experiments

To test the adsorption strength of the O atoms involved in these structures, we performed a series of experiments in which we evacuated the reactor after having formed the oxygen-rich structures in situ at high temperature and high O_2 pressure. The decrease in pressure was rapid and the pressure reached the (U)HV-regime within minutes, at which point the STM imaging was resumed. At the same time, heating of the surface was stopped and the sample was allowed to cool down.

The results obtained are summarized in Supplementary Fig. 6. The stable structures formed at 1.0 and 5.0 bar O_2 are illustrated by the STM images in Supplementary Figs. 6a and d, respectively. In both images the spoked-wheel oxide can be distinguished. In addition, the higher pressure exposure resulted in a large fraction of the surface covered with the lifted-row structure. After growth, the reactor was evacuated and two representative STM images are shown in Supplementary Figs. 6b and e. They show that both well-ordered structures were unstable in the absence of the O_2 atmosphere. However, some hints of the remaining structures could still be distinguished. Some of these vague structures are highlighted in Supplementary Fig. 6c.

After evacuation, the imaged surface appeared very rough and the STM signal much noisier. This increase in noise under (U)HV conditions compared to high-pressure conditions seems counterintuitive. To explain this, we propose that both structures were thermodynamically unstable without the high oxygen pressure. These unstable structures were releasing O_2, which increased the mobility of Pt atoms. This mobility is then responsible for the observed noise in the tunneling current, which affected the quality of the STM images and was observed as single-scan-line noise. Under these conditions, we saw evidence for a significant influence of the tip on the surface structure, possibly via its interaction with the mobile Pt atoms or by enhancing the rate of departure of O atoms. For example, the STM image of Supplementary Fig. 6f was recorded after the central region of the image had been scanned several times, and it showed that this region had suffered a loss of material with respect to its surroundings, while the stripiness indicates significant mobility in or on the depressed region.

There was a clear difference between the surface obtained after evacuation, starting from 1.0 bar O_2 and that from 5.0 bar O_2. After exposure to 1.0 bar O_2 and subsequent evacuation, the terraces exhibited a noisy two-level structure (Supplementary Fig. 6f) that was less well organized than the two-level pattern of Fig. 2b. The STM image following evacuation after exposure to 5.0 bar O_2 showed that the surface tried to avoid steps perpendicular to the lifted oxide rows (Supplementary Fig. 6e). The observed step configurations are reminiscent of those on the missing-row-reconstructed Pt(110) surface[14,15].

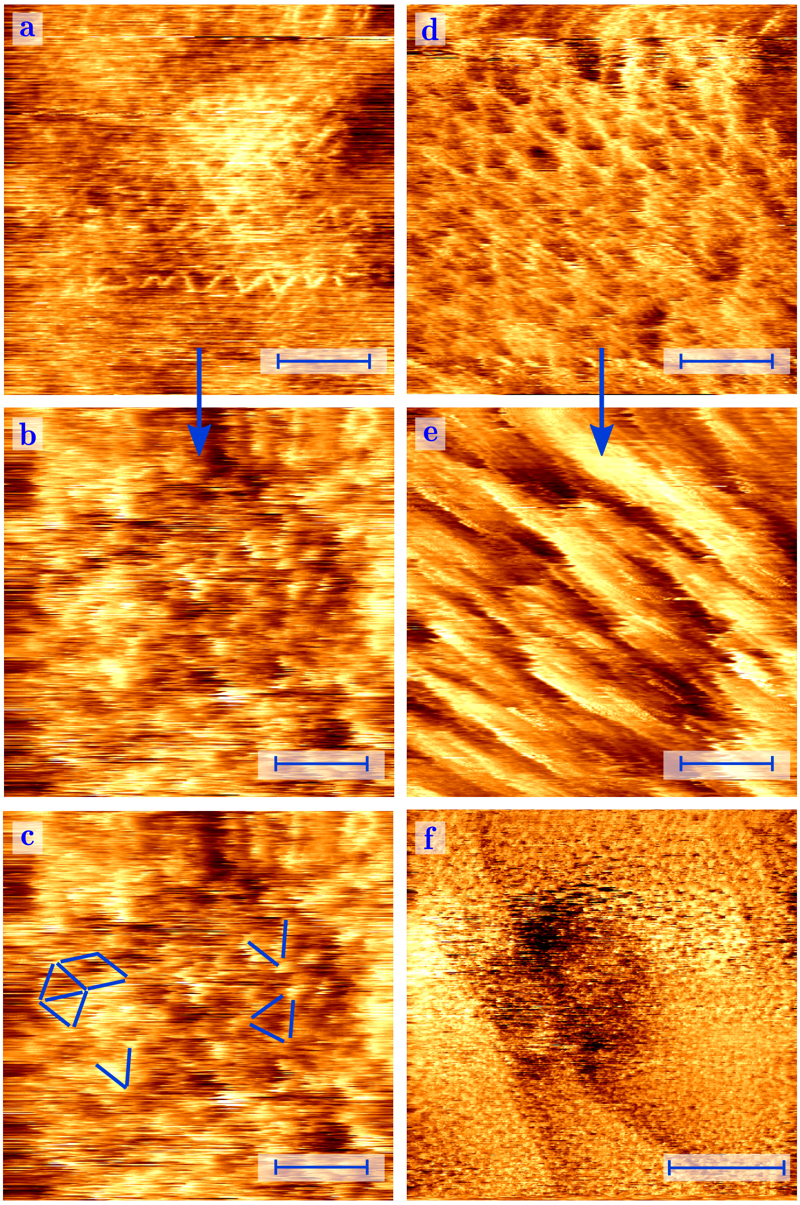

Supplementary Fig. 6: Structure after evacuation. STM images of two separate experiments: images obtained in situ, while oxidizing the surface, either at 1.0 bar (a) or 5.0 bar O_2 (d), after which the reactor was evacuated (b, c, f, following a, and e, following d). Under oxidizing conditions, surface is mainly covered with spoked wheels (a, p_{O_2} = 1.0 bar, T_{sample} = 529 K, t_{exposure} = 45 minutes, 25×25 nm^2, U_{bias} = 0.05 V, $\overline{I_{\text{tunnel}}}$ = −2.66 nA, and t_{image} = 17.8 s). Image b, disordered structure, obtained 57 minutes after evacuation, (T_{sample} = 336 K, 25×25 nm^2, U_{bias} = 0.05 V, $\overline{I_{\text{tunnel}}}$ = −0.48 nA, and t_{image} = 17.8 s). Image c (replica of b) highlights several elements that hint at residues of the spoked-wheel structure. At 5.0 bar O_2, a mixture of spoked wheels and lifted rows formed (d, T_{sample} = 538 K, t_{exposure} = 162 minutes, 25×25 nm^2, U_{bias} = 0.1 V, $\overline{I_{\text{tunnel}}}$ = −0.95 nA, t_{image} = 15.1 s). Image e, 35 minutes after evacuation, vague row pattern still discernible and many steps following the rows, T_{sample} = 353 K (25×25 nm^2, U_{bias} = 0.1 V, $\overline{I_{\text{tunnel}}}$ = −0.71 nA, and t_{image} = 18.3 s). Image f, 71 minutes after evacuation, the surface is susceptible to tip-induced restructuring indicated by a changed morphology in the center of the STM image, T_{sample} = 331 K (200 × 200 nm^2, U_{bias} = −0.03 V, $\overline{I_{\text{tunnel}}}$ = 0.26 nA, and t_{image} = 75.8 s). Scale bars represent 6 nm (a–e) and 60 nm (f).

Supplementary Table 3: XPS, O(ads). Reported values in literature of O 1s binding energies attributed to chemisorbed O on Pt.

O 1s B.E. / eV	Sample	Reference	Notes
529.4	Pt(111)	16	
529.5	Pt(531)	17	
529.55	Pt(332)	18	
529.7	Pt(111)	19	
529.7	Pt(110)	20	
529.75	Pt(111)	18	
529.8	Pt(111)	21	
530.2	Pt(111)	22	
530.8	Pt(111)	23	[1]
530.8	Pt(111)	24	
530.8	Pt(100)	24	

[1] After decomposition of Pt oxide.

Supplementary Table 4: XPS, oxidic. Reported values in literature of O 1s binding energies assigned to oxidized Pt.

O 1s B.E. / eV	Assignment	Reference	Notes
529.5	surface oxide	19	Pt(111)
530.1	PtO_2	19	Pt(111)
530.2	PtO_2 on Pt	22	Pt(111)
530.3	PtO_2 (bulk)	22	Pt(111)
530.3	PtO_2	25	[1]
530.4	PtO_2	26	[2]
530.5	oxidic layer	23	[3]
530.6	PtO_{1+x}	27	[1]
530.6	PtO_2	27	[1]
530.8	surface oxide	17	Pt(531)
530.8	surface oxide	20	Pt(110)
531	PtO	27	[1], broad
531.4	PtO	27	[1]

[1] Sputtered Pt oxide films.
[2] After O_2-plasma exposure of polycrystalline Pt film.
[3] Fitted with peaks at 530.8 eV (chem. O) and 530.2 eV (oxide).

Supplementary Note 7: Temperature-dependent XPS study

A second X-ray photoelectron spectroscopy study focused on the temperature dependence of the spoked-wheel structure. The XP spectra were taken at temperatures between 432 and 963 K. The results are presented in Supplementary Fig. 7. The spectrum labeled "1056 K" was collected at lower temperatures after annealing briefly to 1056 K. The change in O coverage plotted as function of temperature is depicted in Supplementary Fig. 7b. The graph shows a constant, almost linear decrease in O coverage with increasing temperature. After annealing to 1056 K, the O coverage was on the level of the experimental noise, as is indicated by the large error margin in the binding energy for that temperature.

In the first (black) spectrum in Supplementary Fig. 7, some intensity was observed around 535 eV. It was not clear whether this was a real peak, noise or a change in background. This was not observed in the higher resolution spectrum of Fig. 4b (main text).

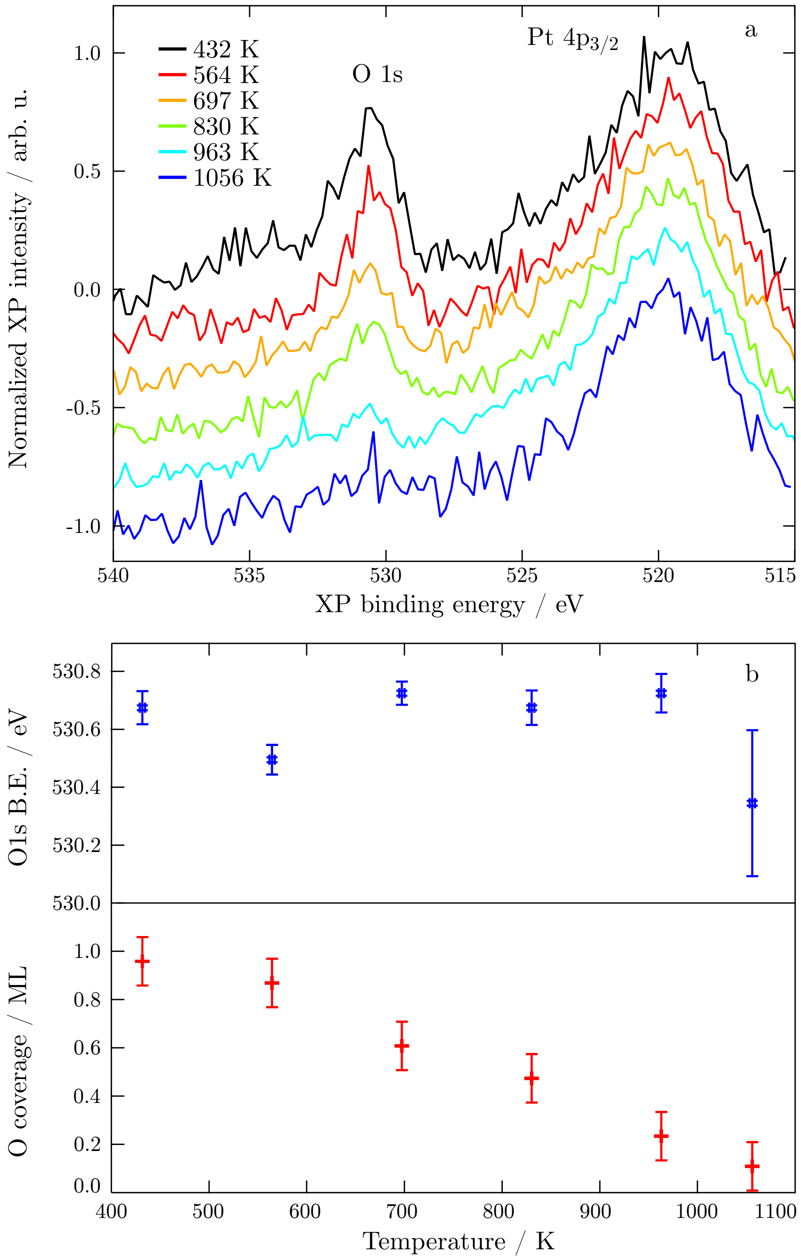

Supplementary Fig. 7: Temperature-programmed XPS. XP spectra (a) measured at different sample temperatures after high-pressure and high-temperature exposure (1.0 bar O_2, 433–438 K, 53 minutes, after which the sample was allowed to cool to 332 K in 33 minutes at the same O_2 pressure). Temperature dependence of (b) the fitted binding energies of the O 1s peak (blue number signs; upper panel) and the integrated O 1s peak areas (red pluses; lower panel). The error bars were based on the uncertainty (standard deviation) of the binding energy in the fit. Lower left axis, integrated O 1s peak areas plotted as a function of temperature (red crosses). Error bars were based on the variation of coverage with changes in fitting parameters. This was estimated to be ± 0.1 ML.

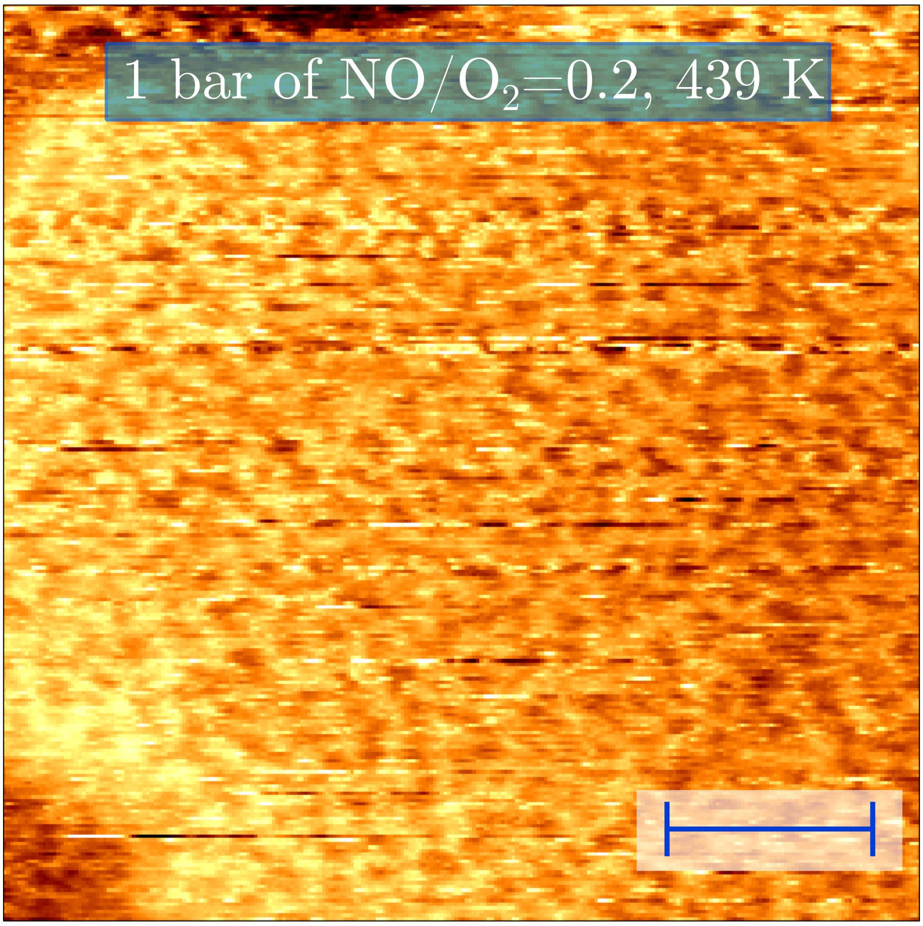

Supplementary Fig. 8: Pt(111) under NO oxidation conditions. Large-scale STM image (100×100 nm^2) depicting the network of islands formed on the surface of Pt(111) in a mixture of NO/O_2=0.2 at a pressure of 1.0 bar at 439 K. $U_{\text{bias}}=0.05$ V, $\overline{I_{\text{tunnel}}}=-0.2$ nA. Scale bar represent 22 nm.

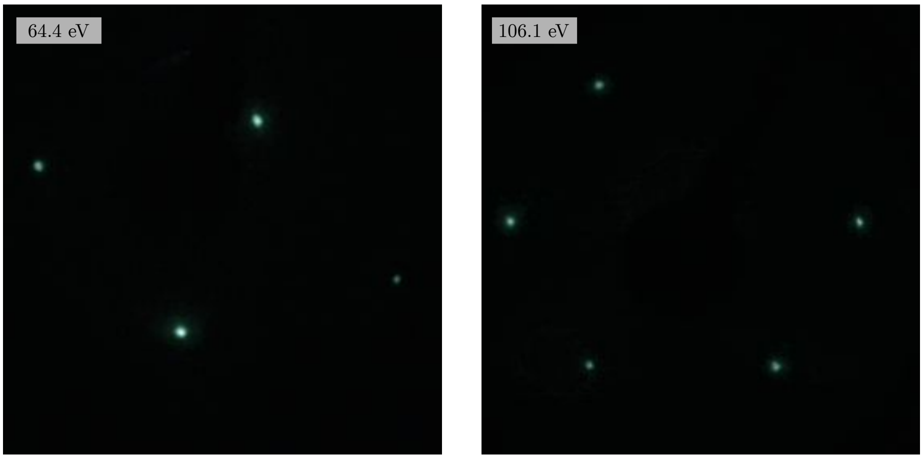

Supplementary Fig. 9: Low-energy electron diffraction patterns. Two photographs of LEED patterns from a freshly prepared Pt(111) surface taken at an electron energy of 64.4 (left) and 106.1 eV (right).

Supplementary References

1. Devarajan, S. P., Hinojosa Jr, J. A. & Weaver, J. F. STM Study of High-Coverage Structures of Atomic Oxygen on Pt(1 1 1): p(2×1) and Pt Oxide Chain Structures. *Surf. Sci.* **602**, 3116–3124 (2008).

2. Weaver, J. F. Surface Chemistry of Late Transition Metal Oxides. *Chem. Rev.* **113**, 4164–4215 (2013).

3. Moore, W. J. & Pauling, L. The Crystal Structures of the Tetragonal Monoxides of Lead, Tin Palladium, and Platinum. *J. Am. Chem. Soc.* **63**, 1392–1394 (1941).

4. Galloni, E. E. & Busch, R. H. The Structure of Platinum Oxides. *J. Chem. Phys.* **20**, 198–199 (1952).

5. Shishakov, N. A., Asanov, U. & Andrushchenko, N. K. Simple Method of Preparing Pure Platinum Dioxide. *Russ. Chem. Bull.* **9**, 350–351 (1960).

6. Muller, O. & Roy, R. Formation and Stability of the Platinum and Rhodium Oxides at High Oxygen Pressures and the Structure of the Pt_3O_4, β-PtO_2 and RhO_2. *J. Less-Common Metals* **16**, 129–146 (1968).

7. McBride, J. R., Graham, G. W., Peters, C. R. & Weber, W. H. Growth and Characterization of Reactively Sputtered Thin-Film Platinum Oxides. *J. Appl. Phys.* **69**, 1596–1604 (1991).

8. Westwood, W. D. & Bennewitz, C. D. Formation of PtO Films by Reactive Sputtering. *J. Appl. Phys.* **45**, 2313–2315 (1974).

9. Galloni, E. E. & Roffo Jr, A. E. The Crystalline Structure of Pt_3O_4. *J. Chem. Phys.* **9**, 875–877 (1941).

10. Cahen, D., Ibers, J. A. & Wagner Jr, J. B. Platinum Bronzes. IV. Preparation, Crystal Chemistry, and Physical Properties. *Inorg. Chem.* **13**, 1377–1388 (1974).

11. Graham, G. W., Weber, W. H., McBride, J. R. & Peters, C. R. Raman Investigation of Simple and Complex Oxides of Platinum. *J. Raman Spectrosc.* **22**, 1–9 (1991).

12. Saenger, K. L., Cabral Jr, C., Lavoie, C. & Rossnagel, S. M. Thermal Stability and Oxygen-Loss Characteristics of Pt(O) Films Prepared by Reactive Sputtering. *J. Appl. Phys.* **86**, 6084–6087 (1999).

13. Wang, C.-B., Lin, H.-K., Hsu, S.-N., Huang, T.-H. & Chiu, H.-C. Enthalpies of Reduction–Oxidation of Alumina-Supported Platinum. *J. Mol. Catal. A: Chem.* **188**, 201–208 (2002).

14. Van Spronsen, M. A., Van Baarle, G. J. C., Herbschleb, C. T., Frenken, J. W. M. & Groot, I. M. N. High-pressure Operando STM Studies Giving Insight in CO Oxidation and NO Reduction over Pt(1 1 0). *Catal. Today* **244**, 85–95 (2015).

15. Van Spronsen, M. A., Van Baarle, G. J. C., Herbschleb, C. T., Frenken, J. W. M. & Groot, I. M. N. Erratum to "High-Pressure Operando STM Studies Giving Insight in CO Oxidation and NO Reduction over Pt(1 1 0)" [Catal. Today 244 (2015) 85–95]. *Catal. Today* **256, Part 2**, 384 (2015).

16. Björneholm, O. *et al.* Overlayer Structure from Adsorbate and Substrate Core Level Binding Energy Shifts: CO, CCH_3 and O on Pt(111). *Surf. Sci.* **315**, L983–L989 (1994).

17. Held, G., Jones, L. B., Seddon, E. A. & King, D. A. Effect of Oxygen Adsorption on the Chiral Pt{531} Surface. *J. Phys. Chem. B* **109**, 6159–6163 (2005).

18. Wang, J. G. *et al.* One-Dimensional PtO_2 at Pt Steps: Formation and Reaction with CO. *Phys. Rev. Lett.* **95**, 256102 (2005).

19. Miller, D. J. *et al.* Oxidation of Pt(111) under Near-Ambient Conditions. *Phys. Rev. Lett.* **107**, 195502 (2011).

20. Butcher, D. R. *et al.* In Situ Oxidation Study of Pt(110) and Its Interaction with CO. *J. Am. Chem. Soc.* **133**, 20319–20325 (2011).

21. Gland, J. L. Molecular and Atomic Adsorption of Oxygen on the Pt(111) and Pt(S)-12(111)×(111) Surfaces. *Surf. Sci.* **93**, 487–514 (1980).

22. Peuckert, M. & Bonzel, H. P. Characterization of Oxidized Platinum Surfaces by X-Ray Photoelectron Spectroscopy. *Surf. Sci.* **145**, 239–259 (1984).

23. Parkinson, C. R., Walker, M. & McConville, C. F. Reaction of Atomic Oxygen with a Pt(111) Surface: Chemical and Structural Determination Using XPS, CAICISS and LEED. *Surf. Sci.* **545**, 19–33 (2003).

24. Derry, G. N. & Ross, P. N. High Coverage States of Oxygen Adsorbed on Pt(100) and Pt(111) Surfaces. *Surf. Sci.* **140**, 165–180 (1984).

25. Abe, Y., Yanagisawa, H. & Sasaki, K. Preparation of Oxygen-Containing Pt and Pt Oxide Thin Films by Reactive Sputtering and Their Characterization. *Jpn. J. Appl. Phys.* **37**, 4482–4486 (1998).

26. Blackstock, J. J., Stewart, D. R. & Li, Z. Plasma-Produced Ultra-Thin Platinum-Oxide Films for Nanoelectronics: Physical Characterization. *Appl. Phys. A* **80**, 1343–1353 (2005).

27. Hecq, M., Hecq, A., Delrue, J. P. & Robert, T. Sputtering Deposition, XPS and X-Ray Diffraction Characterization of Oxygen-Platinum Compounds. *J. Less-Common Met.* **64**, P25–P37 (1979).
