# Supplementary material for: Observing the oxidation of platinum
Source: Nat Commun. 2017 Sep 5;8:429. doi: 10.1038/s41467-017-00643-z (PMC5585323; doi:10.1038/s41467-017-00643-z)
Supplement: Supplementary file 2 — Peer Review File [file 41467_2017_643_MOESM2_ESM.pdf]

Editorial Note: this manuscript has been previously reviewed at another journal that is not operating a transparent peer review scheme. This document only contains reviewer comments and rebuttal letters for versions considered at Nature Communications.
